# Does Listening to Music Improve Pain Perception and Anxiety in Patients Undergoing Cystoscopy: A Meta-Analysis

**DOI:** 10.3389/fsurg.2021.689782

**Published:** 2021-06-28

**Authors:** Guo Chen, Cai Tang, Yuebai Liu, Yuhao Liu, Yi Dai, Luo Yang

**Affiliations:** ^1^Department of Urology, West China Fourth Hospital of Sichuan University, Chengdu, China; ^2^Laboratory of Reconstructive Urology, Institute of Urology, West China Hospital, Sichuan University, Chengdu, China; ^3^Department of Education and Training, Sichuan Cancer Hospital, Chengdu, China

**Keywords:** anxiety, cystoscopy, music, pain, effect

## Abstract

**Objective:** To identify the effect of music on outpatient-based cystoscopy.

**Methods:** We systematically reviewed the effect of music on all reported outpatient for cystoscopy and extracted data from randomized trials from inception to February 3, 2021, with no language restrictions. The analysis was completed *via* STATA version 14.2.

**Results:** A total of 27 studies were initially identified, and 6 articles containing 639 patients were included in the final analysis. In terms of post-procedural pain perception, a pooled analysis of 6 articles containing 639 patients showed that music seems to improve discomfort in patients who undergo cystoscopy (WMD: −1.72; 95%CI: −2.37 to −1.07). This improvement remained consistent in patients undergoing flexible cystoscopy (FC) (WMD: −1.18; 95% CI: −1.39 to −0.98) and rigid cystoscopy (RC) (WMD: −2.56; 95% CI: −3.64 to −1.48). The music group also had less post-procedural anxiety than those in no music group during cystoscopy (WMD: −13.33; 95% CI: −21.61 to −5.06), which was in accordance with the result of FC (WMD: −4.82; 95% CI: −6.38 to −3.26) than RC (WMD: −26.05; 95% CI: −56.13 to 4.04). Besides, we detected a significantly lower post-procedural heart rate (HR) in the music group than no music group during cystoscopy (WMD: −4.04; 95% CI: −5.38 to −2.71), which is similar to the results of subgroup analysis for FC (WMD: −3.77; 95% CI: −5.84 to −1.70) and RC (WMD: −4.24; 95% CI: −5.98 to −2.50). A pooled analysis of three trials indicated that patients in the music group had significantly higher post-operative satisfaction visual analog scale (VAS) scores than those in the no-music group during RC. However, there was no significant difference between the music group and no music group regarding post-procedural systolic pressures (SPs) during cystoscopy (WMD: −3.08; 95% CI: −8.64 to 2.49). For male patients undergoing cystoscopy, the music seemed to exert a similar effect on decreasing anxiety and pain, and it might serve as a useful adjunct to increase procedural satisfaction.

**Conclusions:** These findings indicate that listening to music contributes to the improvement of pain perception, HR, and anxiety feeling during cystoscopy, especially for male patients undergoing RC. Music might serve as a simple, inexpensive, and effective adjunct to sedation during cystoscopy.

## Introduction

Cystoscopy is well-recognized as one of the most frequent procedures in outpatient urology, which is almost performed under local anesthesia. Urologists usually recommend their patients for a cystoscopy to figure out the etiology of lower urinary tract symptoms, such as hematuria, suspicious bladder tumor, and recurrent urinary tract infections (1, 2). However, the feelings of pain and anxiety discourage many patients from undergoing this clinical examination (2). For the past decades, clinical practitioners have tried various methods to alleviate the suffering of patients, such as parenteral agents (3), inhalational agents (4), intraurethral lidocaine (5), watching relaxing video (6, 7), bag squeeze (8), and flexible cystoscopy (FC) (9), but it can be difficult to achieve complete pain relief during cystoscopy under local anesthesia.

Music therapy was established as an adjuvant treatment to be beneficial in a variety of clinical settings including dental extractions, coronary heart disease, during colonoscopy, and prostate biopsies (10–13). Currently, an increasing number of original articles have been developed to explore its potentiality in cystoscopy. Despite two previous reviews (14, 15) showing a beneficial effect of music on urologic outpatient cystoscopy, the evidence remains underpowered. The present study aimed to evaluate the efficacy of music as an adjunct to routine local anesthesia in reducing pain and anxiety in patients undergoing cystoscopy.

## Methods

### Search Strategy

In accordance with the preferred reporting items for systematic review and meta-analysis (PRISMA) guidelines (16), we conducted a systematic literature search by using PubMed, the Cochrane Library, and Embase using the search terms of “music” and “cystoscopy” before February 3, 2021, without any language limitations to identify possible studies.

### Study Selection

**Figure 1:**
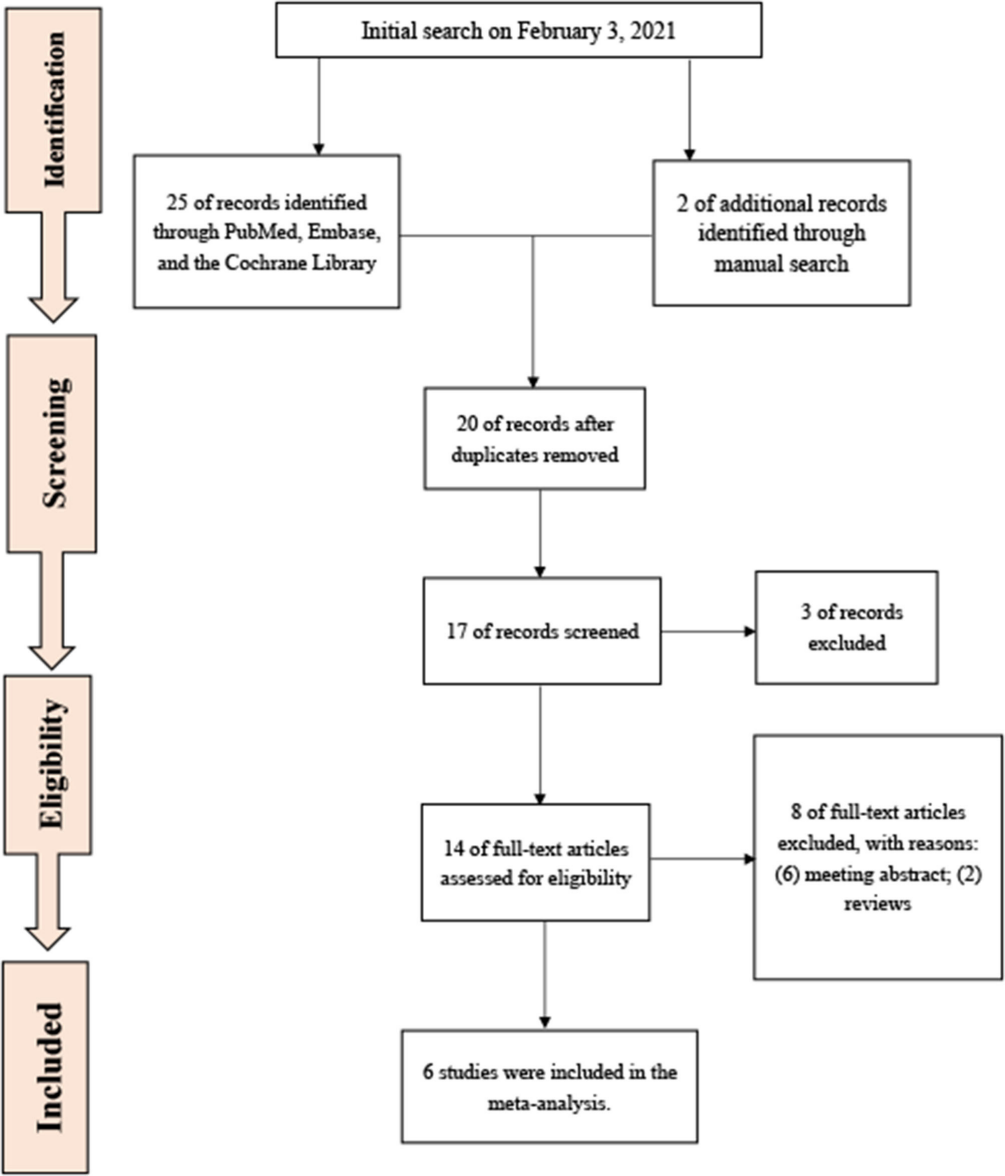
The preferred reporting items for systematic review and meta-analysis (PRISMA) flowchart.

We used the PICOS method to define the inclusion criteria. Patients (P): patients undergoing FC or rigid cystoscopy (RC); intervention (I): listening to music during cystoscopy; comparison (C) of publications to compare music with no music; outcomes (O): post-procedural pain perception measured by visual analog scale (VAS) ranging from 0 to 10 (17). High scores on the scale indicate that pain intensity is high. Post-procedural anxiety levels were measured by the State-Trait Anxiety Inventory (STAI) questionnaire (18), which consists of 20 questions. STAI scores range from 20 to 80, and a higher score indicated a greater anxiety level. Post-procedural satisfaction was assessed by VAS, and a higher VAS score represented a greater satisfaction level. Post-procedural heart rate (HR) or the pulse and systolic pressure (SP) were also measured. Study design (S): The included studies were all randomized controlled trials (RCTs). Exclusion criteria included the following items: (1) meeting abstracts and reviews including meta-analysis; (2) no systemic sedation or analgesia before cystoscopy; (3) current urinary infection; (4) presence of current pain in the pelvic region (e.g., a bladder pain syndrome or interstitial cystitis); (5) pregnancy; (6) prior urethral surgery; (7) cystoscopy with other interventions; and (8) data not available. Figure 1 shows the flowchart of the selection process of the study.

### Data Extraction and Quality Assessment

Two authors were independently involved to screen the search results in the following order to remove duplicates, title, abstract, and finally full text according to prespecified standardized criteria. Disagreements were resolved by discussion. Two independent reviewers used the preformulated tables to extract data. The following data were extracted: the name of the first author, year of publication, country, period, age, sample size, music type, local anesthesia, and cystoscopy type.

Two independent authors evaluated the methodological quality of the studies according to the Cochrane Collaboration's risk of bias (RoB) tool in Review Manager software (19). This tool primarily evaluates 7 domains: random sequence generation (selection bias); allocation concealment (selection bias); blinding of participants and personnel (performance bias); blinding of outcome assessment (detection bias); incomplete outcome data (attrition bias); selective reporting (reporting bias); and other bias (such as funding sources). In addition, two reviewers independently rated the level of evidence (LoE) of the included articles through the Oxford Centre for Evidence-Based Medicine criteria (20); This scale graded studies from strongest (level 1) to weakest (level 5) strength of evidence according to the study design and data quality.

**Figure 2:**
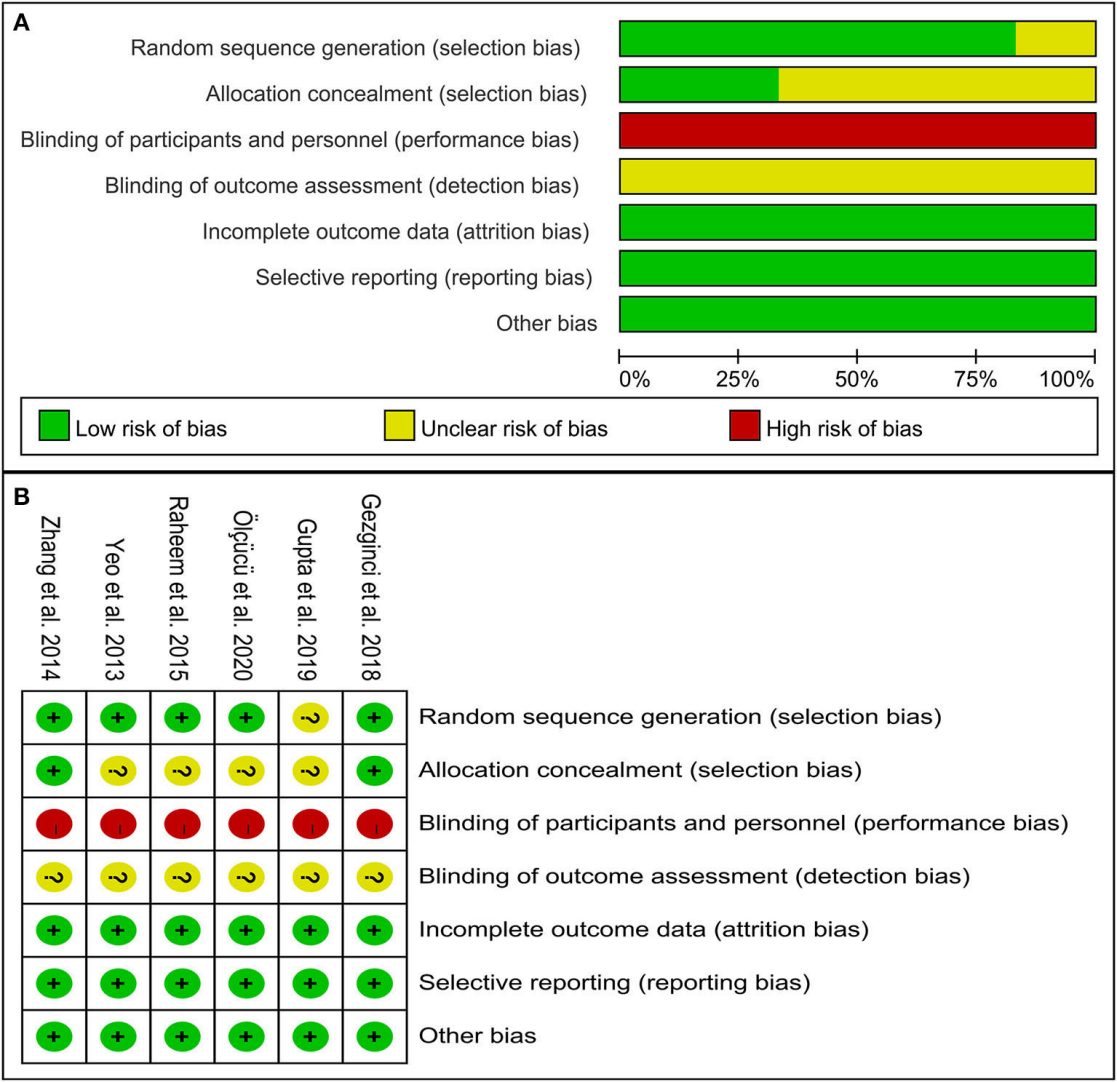
The methodological quality of studies included in this study. **(A)** Risk of bias graph. **(B)** Risk of bias summary.

Figure 2 shows the RoB summary of the six RCTs (21–26). Overall, included studies had a low risk of selection bias, attrition bias, and reporting bias. However, the risk of performance bias was high. The risk of detection bias was unclear due to the absence of related descriptions.

### Statistical Analysis

The continuous variables were described as means ± SD. Median and range were used to estimate mean and SD (27). The percentiles, 25th, and 75th percentiles as well as 5th and 95th percentiles, were transformed to SD by using the following formula: SD ≈ Norm IQR = (P75−P25) × 0.7413 (IQR: inter-quartile range, P75: 75th percentile, P25: 25th percentile) (28). Mean difference (MD) was pooled for continuous variables. The fixed-effects model was used unless there exists heterogeneity (I^2^ > 50%, *p* < 0.1), and the level of significance was set at *p* < 0.05. Additionally, we performed a subgroup analysis based on gender. Besides, we conducted subgroup analyses for types of cystoscopy and male patients. This meta-analysis was completed by STATA version 14.2.

## Results

### Search Results

A total of 27 studies were initially identified, and 6 articles (21–26) containing 639 patients were included in the final analyses. The participants in this meta-analysis were from China, the USA, Turkey, Korea, and India. Four of six studies (21, 23–25) enrolled only male patients, and three of six studies (21–23) used FC. Five of six studies (21–25) followed intraurethral administration of 2% lidocaine jelly prior to cystoscopy, and only one study (26) made use of 2% xylocaine jelly before RC to conduct local anesthesia. Table 1 shows the main characteristics of studies included in this meta-analysis.

**Table 1:** The main characteristics of included studies in this meta-analysis.

**Study**	**Country**	**Period**	**Gender**	**Age (years)**	**Music**	**Local anesthesia**	**Cystoscopy**	**LoE**
Zhang et al. (21)	China	2013.1to2013.9	Male: 124	Mean (SD) EG: 64.8 (11.2) CG: 62.0 (12.7)	Classical music, Chinese folk music, popular music, and foreign music ready for patient selection	10 ml 2% lidocaine jelly administrated intraurethrally for 3 min before FC	16F FC (Olympus CYF-5A)	2b
Raheem et al. (22)	USA	2011.6–2013.6	Male: 129 Female: 8	Mean (SD) EG: 65.8 (9.9) CG: 67.1 (10.4)	Classical music	10 ml 2% lidocaine jelly administrated intraurethrally for 15 min before FC	15F FC (Olympus CYF-5A)	2b
Ölçücü et al. (23)	Turkey	2019.7–2020.3	Male: 148	Mean (SD) EG: 57.89 (12.71) CG: 55.56 (16.41)	Classical music	10 ml 2% lidocaine jelly administrated intraurethrally for 5 min before FC	17F FC (Hawk GmbH, China)	2b
Yeo et al. (24)	Korea	2011.5–2011.12	Male: 70	Mean (SD) EG: 47.3 (15.3) CG: 49.1 (13.5)	Classical music	10 ml 2% lidocaine jelly administrated intraurethrally for 15 min before FC	RC	2b
Gezginci et al. (25)	Turkey	2016.3–2017.3	Male: 60	Mean (range) EG: 58.5 (21.0–80.0) CG: 64.0 (22.0–83.0)	Turkish folk music, Turkish art music, Turkish arabesque music, Turkish pop music, foreign pop music, rock music, and/or classical music	10 ml 2% lidocaine jelly administrated intraurethrally for 15 min before RC	19F RC	2b
Gupta et al. (26)	India	2017.4–2017.9	Male: 63 Female: 38	Mean (SD) EG: 53.68 (8.60) CG: 52.08 (10.04)	Not reported	2% xylocaine jelly administrated intraurethrally for 10 min before RC	17.5F RC	2b

*SD, standard deviation; LoE, level of evidence; RC, rigid cystoscopy; FC, flexible cystoscopy; EG, experimental group; CG, control group*.

### Meta-Analysis Results

**Figure 3:**
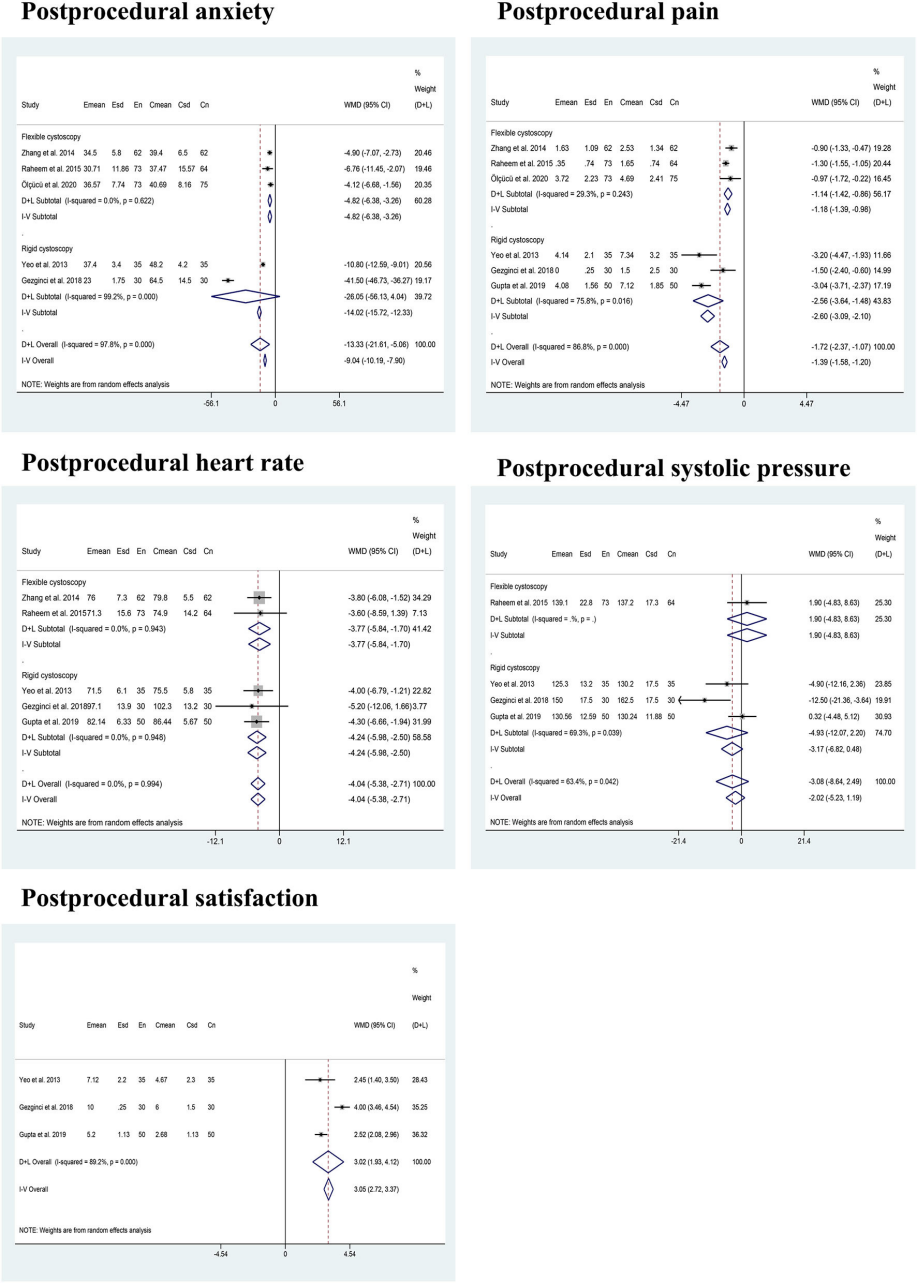
The pooled results of the evaluated outcomes in this study.

In terms of post-procedural pain perception, a pooled analysis of six articles (21–26) containing 639 patients showed that patients listening to music experienced less significant discomfort than their counterparts during cystoscopy (WMD: −1.72; 95% CI: −2.37 to −1.07). This improvement remained consistent in patients undergoing FC (WMD: −1.18; 95% CI: −1.39 to −0.98) and RC (WMD: −2.56; 95% CI: −3.64 to −1.48). Patients in the music group also had less post-procedural anxiety feeling than those in no music group during cystoscopy (WMD: −13.33; 95% CI: −21.61 to −5.06), which was in accordance with the result of FC (WMD: −4.82; 95% CI: −6.38 to −3.26) than RC (WMD: −26.05; 95% CI: −56.13 to 4.04). Besides, we detected a significantly lower post-procedural HR in the music group than no music group during cystoscopy (WMD: −4.04; 95% CI: −5.38 to −2.71), which is similar to the results of subgroup analysis for FC (WMD: −3.77; 95% CI: −5.84 to −1.70) and RC (WMD: −4.24; 95% CI: −5.98 to −2.50). A pooled analysis of three trials (24–26) indicated that patients in the music group had significantly higher post-operative satisfaction VAS scores than those in the no music group during RC. However, there was no significant difference between the music group and the no music group regarding post-procedural SP during cystoscopy (WMD: −3.08; 95% CI: −8.64 to 2.49). Figure 3 presents the meta-analysis results of estimated outcomes.

### Subgroup Analysis Results of Male Patients

**Figure 4:**
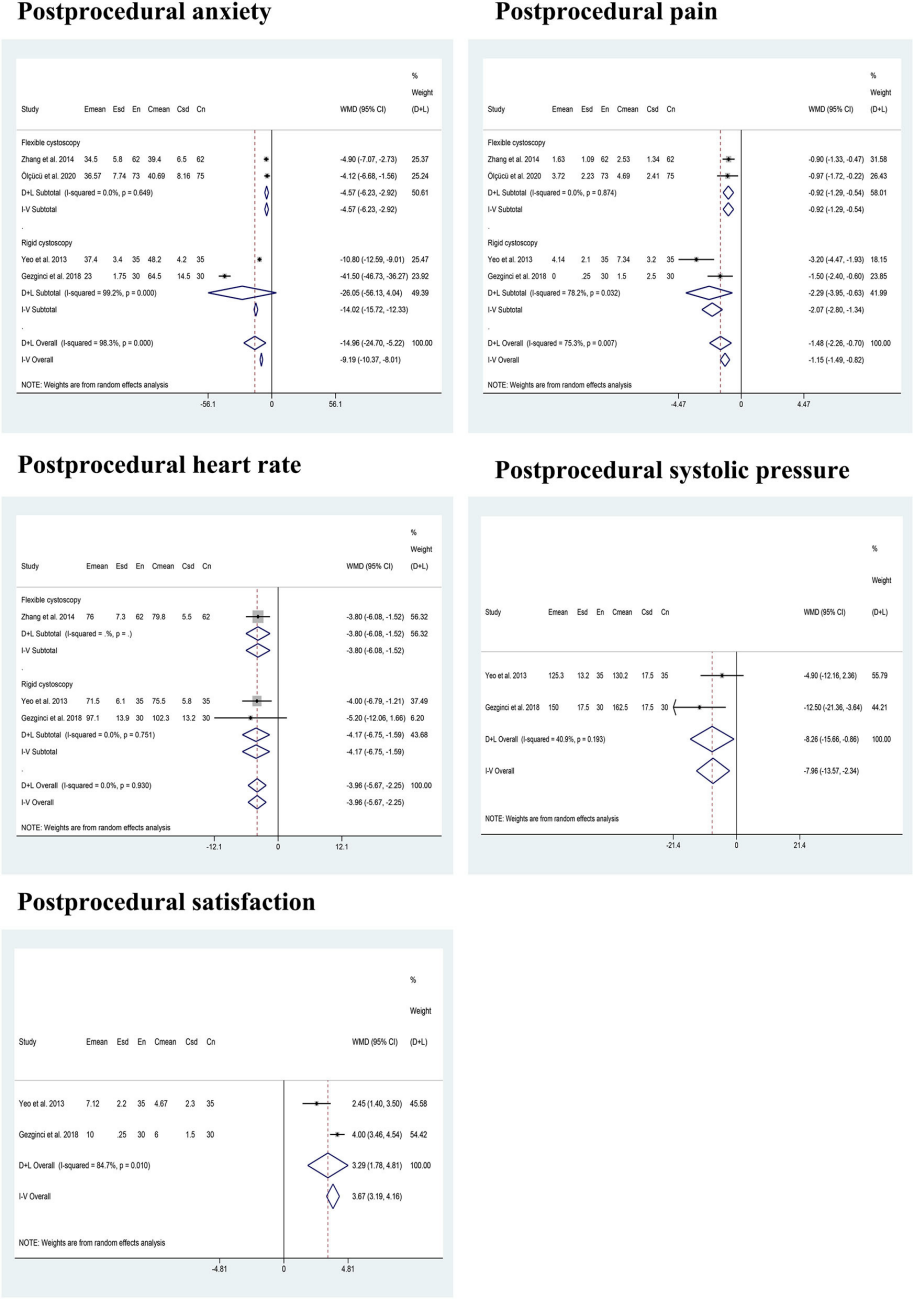
The results of the subgroup analysis in this study.

A pooled analysis of four articles (21, 23–25) including 402 male participants showed that there was significantly less post-procedural pain perception for patients in the music group than those in the no music group (WMD: −1.48; 95% CI: −2.26 to −0.70). These results were coincidentally stratified by FC (WMD: −0.92; 95% CI: −1.29 to −0.54) and RC (WMD: −2.29; 95% CI: −3.95 to −0.63). Four articles (21, 23–25) reported post-procedural anxiety. Meta-analysis showed that patients listening to music had significantly less anxiety feeling compared with the control group (CG) (WMD: −14.96; 95% CI: −24.70 to 5.22), but no difference was identified between the two groups of patients undergoing RC (WMD: −26.05; 95% CI: −56.13 to 4.04). A pooled analysis of three articles (21, 24, 25) showed that lower post-procedural HR was identified in the music group compared with the no music group during cystoscopy (WMD: −3.96; 95% CI: −5.67 to −2.25), as well as during RC (WMD: −4.17; 95% CI: −6.75 to −1.59). Besides, a pooled analysis of two studies (24, 25) showed that patients in the music group experienced significantly higher post-operative satisfaction VAS scores (WMD: 3.29; 95% CI: 1.78 to 4.81) and lower post-procedural SPs (WMD: −7.96; 95% CI: −13.57 to −2.34) compared with those in the no music group. Figure 4 presents the results of subgroup analysis of male patients.

## Discussion

In many urological institutions, an intraurethral instillation of 2% lidocaine gel is often required to provide local anesthesia and to reduce pain before cystoscopy (5, 29). However, cystoscopy is invasive and might lead to increased pain perception and anxiety levels, despite local anesthesia. Patients having high anxiety levels usually experience longer pain and require more analgesics (30, 31), because anxiety is frequently related to increased pain perception (32, 33). In the present study, we found that music intervention was significantly associated with decreased pain perception, anxiety, and HR levels. Furthermore, this improvement was more pronounced for patients undergoing RC, which was consistent with previous studies (9, 34, 35). They observed that FC was more tolerable than RC, especially in male patients (9, 35). Besides, patients in the music group had higher satisfaction VAS scores than those in the CG during RC.

It is well-known that there are anatomical gender differences with respect to urethra (36). Males have prostate, tight sphincter, and longer urethra (23, 36). Thus, male patients suffer more painful experiences than females (21, 23). Given this, we also conducted a subgroup analysis for male patients undergoing cystoscopy. We demonstrated a beneficial effect of music on pain perception, anxiety levels, HR, and SP. Furthermore, male patients undergoing RC seem to benefit more and are more satisfied with music intervention as well. Compared to other systematic reviews, the present review performed a subgroup analysis of gender and added three new studies updating the evidence level.

The present study does have some limitations. First, the limited number of studies, sample sizes, the study population, the experience of the surgeon, and the definition of outcome measures make it difficult to emphatically confirm the advantage of music intervention. Besides, the ethnic differences in penis and diameters of FC might affect the results. Second, the surgeons were not blinded during the procedure, so it is potentially possible that patients in the music group could get more careful manipulation than those in the CG. Finally, VAS and STAI scores are self-reported outcomes of patients, which are partly subjective. We still need to develop more objective parameters and multicenter trials with a large number of patients to yield better results.

## Conclusions

These findings indicate that listening to music contributes to the improvement of pain perception, HR, and anxiety feeling during cystoscopy, especially for male patients undergoing RC. Besides, music might increase procedural satisfaction for male patients undergoing RC as well. Music might serve as a simple, inexpensive, and effective adjunct to sedation during cystoscopy.

## Data Availability Statement

The original contributions presented in the study are included in the article/supplementary material, further inquiries can be directed to the corresponding author/s.

## Author Contributions

LY: conception and design. CT and GC: administrative support. YuhL, GC, and YueL: provision of study materials or patients. YD and YueL: collection and assembly of data. YuhL and GC: data analysis and interpretation. GC: manuscript drafting. All authors: final approval of manuscript.

## Conflict of Interest

The authors declare that the research was conducted in the absence of any commercial or financial relationships that could be construed as a potential conflict of interest.
